# Interpretive autonomy predicts self-regulated learning behaviors in lessons, practice, and performance among classical musicians

**DOI:** 10.3389/fpsyg.2026.1859878

**Published:** 2026-07-10

**Authors:** Marie Fujimoto

**Affiliations:** The Graduate School of Education, The University of Tokyo, Bunkyo-ku, Japan

**Keywords:** interpretive autonomy, music, music education, self-determination theory, self-regulated learning

## Abstract

Although self-regulated learning—planning, monitoring, and evaluating one’s performance to attain personal goals—is vital to improving classical music performance, even advanced musicians struggle to initiate self-regulation in lessons, practice, and performance. While qualitative studies have suggested that autonomy in musical interpretation underpins self-regulated learning behaviors, this has not yet been quantitatively investigated due to the difficulty of measuring interpretive autonomy. Following the model of *Werktreue* internalization, which conceptualizes interpretive autonomy from both psychological (basic psychological need satisfaction in musical interpretation) and behavioral (self-oriented interpretive approaches) aspects, this study conducted a questionnaire survey among 214 student and professional musicians in Japan using newly developed scales. Mediation analysis revealed that basic psychological need satisfaction in musical interpretation predicted the use of self-oriented interpretive approaches, which in turn predicted self-regulated learning behaviors across the lesson, practice, and performance contexts. These findings demonstrate that interpretive autonomy plays a significant role in initiating self-regulated learning behaviors in classical music performance. In addition, by quantifying interpretive autonomy using psychometrics for the first time, this study offers a crucial methodological contribution to a discourse long discussed by qualitative investigations.

## 1 Introduction

Despite years of rigorous training, many advanced classical musicians exhibit suboptimal learning behaviors. Conservatory students often attend lessons without clear objectives, passively following their teachers’ instructions (Gaunt, 2010). During solitary practice, they frequently engage in mindless repetition without having specific aims (McPherson et al., 2019). In performance, musicians often fixate on technical execution rather than being fully immersed in the music; they are then left with feelings of frustration, shame, and hopelessness (Clark et al., 2014).

These learning behaviors may result from a lack of self-regulated learning (SRL; Zimmerman, 2000). Self-regulated learning is defined as “self-generated thoughts, feelings, and actions that are planned and cyclically adapted to the attainment of personal goals” (Zimmerman, 2000, p. 14). This process consists of three phases: forethought, performance, and self-reflection. In the forethought phase, learners analyze the task, set goals, and plan strategies. They also cultivate the motivation necessary to pursue and commit to their goals. In the performance phase, learners strategically direct their focus toward the task and monitor their progress while experimenting with different strategies. In the self-reflection phase, they evaluate the outcome and manage their emotional responses to effectively re-engage in the next cycle of self-regulation.

Researchers have applied the framework of self-regulated learning to examine effective learning behaviors in classical music performance (see Miksza, 2011; Varela et al., 2016; How et al., 2022; Dos Santos Silva and Marinho, 2025 for reviews). While SRL has been primarily applied to the context of individual practice, it is also recognized as a useful framework to investigate learning behaviors in lesson (Fujimoto and Uesaka, 2024, 2025) and performance contexts (Williamon et al., 2017; Fujimoto and Uesaka, 2024, 2025; Lubert and Gröpel, 2024).

However, the factors that contribute to effective self-regulation remain underexplored. Previous studies have identified associations between SRL and demographic variables, such as age, gender, and expertise level; older musicians (Bonneville-Roussy and Bouffard, 2015; Dos Santos Silva et al., 2024), females (Boon, 2020), and professionals (Dos Santos Silva et al., 2024) have been found to demonstrate higher self-regulation skills than younger, male, and student musicians. Regarding psychological aspects, studies have shown that self-efficacy for self-regulation (Miksza and Tan, 2015), self-perceptions of musical competence (Bonneville-Roussy and Bouffard, 2015), and self-efficacy for musical learning (Nielsen, 2004; Ritchie and Williamon, 2013) positively correlate with the use of self-regulation strategies. Yet other psychological constructs need to be explored to fully understand what underpins self-regulated learning behaviors. Given that a lack of self-regulation skills has been observed even among advanced musicians (Dos Santos Silva and Marinho, 2025), understanding the psychological mechanisms that foster self-regulated learning behaviors is essential to advancing our knowledge of musical development and music education.

Therefore, this study aims to offer a new perspective on these contributing factors by investigating how autonomy in musical interpretation plays a pivotal role in initiating self-regulated learning. *Interpretive autonomy* is defined here as the capacity to generate, explore, and integrate interpretive ideas alongside the agency to decide how those ideas are expressed based on one’s own artistic will. Such autonomy is considered fundamental to self-regulated learning; without developing musical ideas, musicians cannot plan how to manipulate performance cues, such as tempo, dynamics, and timbre. Furthermore, they cannot effectively monitor or evaluate how closely their performance communicates their musical intentions. Even when one pursues an interpretation provided by others, if the performer feels pressured to follow it without personal endorsement, the cycle of planning, monitoring, and evaluating to meet an externally imposed goal characterizes other-regulation rather than self-regulation.

Previous studies support that classical musicians who actively develop interpretation demonstrate effective self-regulated learning behaviors in lessons, practice, and performance. For instance, conservatory students who aimed to develop personal interpretations critically evaluated and adopted their teachers’ instructions (Reid, 2001). In practice, musicians who constantly developed interpretive ideas employed diverse practice strategies, such as improvising on the music and creating narratives (Chaffin and Imreh, 2001; Silverman, 2008; Wise et al., 2017; Héroux, 2018; Kegelaers et al., 2022). In performance, musicians reported focusing on musical character and expressive goals during performances they perceived as successful (Clark et al., 2014).

However, developing an original interpretation is neither easy nor simple. Since the nineteenth century, there has been a fundamental norm within the classical music field, known as the *Werktreue* ideology—to be true to the work (Goehr, 1992). This ideology regulates how classical musicians approach interpretation, making interpretation a complex, ill-structured problem; performers are expected to demonstrate originality in how they authentically realize the composer’s intentions. As performance styles considered “authentic” became established throughout the twentieth century, musicologists pointed out that classical musicians often conformed to those performance conventions rather than pursuing their personal understanding of the composer’s intentions. Taruskin (1995, p. 19) argued,

“Where performance-practice research is descriptive, the performance-practice movement is aggressively prescriptive and territorial, dispensing or conferring the status of authenticity as oxymoronical reward for conformity, claiming a specious moral authority, and laying guilt trips on those who fail to endorse its goals.”

This is echoed by Leech-Wilkinson (2020), who observed that classical music is “bedevilled in training and practice by problems of conformity”; “Being able to play anything perfectly, fluently and safely within current performance style is simply a necessary starting-point before artistic virtuosity comes into play” (Chapt. 2). Consonant with this, some conservatory students express anxiety, stemming from the pressure to meet ambiguous yet inviolable standards of authenticity while simultaneously presenting their uniqueness as artists (McCormick, 2015; Hunter and Broad, 2017).

Despite these insights, empirical research remains fragmented; classical musicians hold diverse perspectives on ‘‘authentic’’ interpretation, and this has complicated the conceptualization of interpretive autonomy. Some musicians hold a formalist view, aiming to achieve the fidelity to the composer’s intention by strict adherence to notations on the score (Silverman, 2007). These musicians refrain from imposing their own personality and disregard their own musical ideas if they are not supported by the score (Hultberg, 2002; Héroux, 2016). Others hold a subjective view (Silverman, 2007), seeing the score as an incomplete guide that allows interpretation to be developed based on a musician’s unique feelings and desires. These musicians value the implicit meaning of notations on the score and use narratives or storytelling to personalize the interpretation (Wise et al., 2017; Héroux, 2018). These contrasting views have been found among conservatory students and professional musicians (Hallam, 1995; Hultberg, 2002; Héroux, 2016; Wise et al., 2017), revealing the diverse ideas on what constitute a “faithful” interpretation among classical musicians.

While the formalist view may appear to place performers subservient to the composer (Silverman, 2007), interpretive autonomy needs to be defined independently of beliefs regarding the *Werktreue* ideology. First, a musician’s view cannot be clearly categorized into one or another, as these two views are not necessarily mutually exclusive; Goehr (1992) points out that distinguished conductors often adopt a position of completely committing to realizing the composer’s intentions faithfully while recognizing the importance of developing their own personal interpretation. Moreover, a musician’s self-report can be arbitrary; given that the *Werktreue* ideology is normalized, classical musicians, “if asked, will refer to the default ideology embedded in classical music discourse,” even when the ideology is hidden behind “‘pan-generic’ concerns of presenting a performance for a given audience in a given situation” (Hunter and Broad, 2017, p. 260). In addition, given that a composer’s true intentions are ultimately unknowable, every musician has the need and the right to pursue their own “faithful” interpretation that they truly believe in, however restrictive or liberal it may seem on the surface. Even when they try to eliminate their ego, a musician’s “faithful” interpretive choices reflect their musical preferences and personality in how they generate, explore, and integrate musical ideas, as long as these choices are made autonomously. Therefore, interpretive autonomy should not be defined based on what view a musician takes toward the *Werktreue* ideology, but on how autonomously they adopt that particular view. Similarly, interpretive autonomy should be assessed independently of how closely a musician’s view aligns with interpretive norms. While interpretive norms may be criticized for enforcing conformity, it is possible to autonomously adopt a normative interpretation by fully endorsing its underlying value. However, it has been difficult to distinguish whether one pursues “authenticity” based on their true values and beliefs, or out of anxiety to meet social expectations without finding personal meaning.

To solve this issue, Fujimoto and Uesaka (2024) proposed the model of *Werktreue* internalization which conceptualized interpretive autonomy based on how musicians internalize the *Werktreue* ideology, rather than their views on the *Werktreue* ideology or the extent to which their interpretations deviate from conventional norms. Applying self-determination theory (Deci and Ryan, 2000), their model proposes that musicians internalize the *Werktreue* ideology autonomously when their basic psychological needs for competence, autonomy, and relatedness are satisfied in musical interpretation. Musicians with autonomous *Werktreue* internalization possess interpretive autonomy, as “the *Werktreue* ideology is fully internalized and integrated with the self”; “musicians are freed from concerns of incompetence, pressure, and rejection, and they make interpretive choices based on personal interests, feelings, and intellectual curiosity” (p. 10). Conversely, controlled internalization occurs when these basic psychological needs are thwarted in musical interpretation. Musicians with controlled *Werktreue* internalization lack interpretive autonomy, as “the *Werktreue* ideology remains external to the self”; “musicians are controlled by a sense of incompetence, pressure, and fear of rejection,” and “their self-esteem is contingent on how others evaluate their interpretation, and they are highly self-critical of their interpretation” (p. 10).^1^

The model of *Werktreue* internalization also proposes self-oriented interpretive approaches to address interpretive autonomy through a musician’s attitudes and behaviors in addition to their psychological state. Self-oriented interpretive approaches include six dimensions: reflecting the performer’s personality into the interpretation, finding meaning beyond the notation, selectively adopting teachers’ interpretive ideas, consciously developing an interpretation, spontaneously exploring interpretive ideas, and continuously integrating technique with interpretation (see Fujimoto and Uesaka, 2024 for details).

Based on the model of *Werktreue* internalization, Fujimoto and Uesaka (2025) conducted case studies with eight elite pianists and violinists. The analysis revealed that when musicians felt competent, volitional, and connected in musical interpretation, musicians employed self-oriented interpretive approaches^2^ and demonstrated effective self-regulated learning behaviors. For example, they actively participated in lessons, explored diverse expressive possibilities in practice, and were fully immersed in the music during performance. In contrast, when musicians felt incompetent, forced, and rejected in musical interpretation, musicians did not employ self-oriented interpretive approaches and exhibited dependent learning behaviors; they passively followed teachers’ instructions, only practiced what was told in lessons, and struggled to focus on the music under pressure in performances.

While these previous studies suggest that interpretive autonomy plays a significant role in self-regulated learning, the generalizability of these findings is limited due to the qualitative nature of the research. Therefore, this study aimed to quantitatively investigate how interpretive autonomy predicts self-regulated learning behaviors in lessons, practice, and performance by developing new psychological scales.

This marks the first attempt to measure interpretive autonomy from psychological and behavioral aspects, by the degree of basic psychological need satisfaction in musical interpretation and the use of self-oriented interpretive approaches. These scales can serve as valuable tools to advance empirical studies on interpretive autonomy, which has traditionally been explored in qualitative studies.

A new scale was also developed for self-regulated learning, since existing scales often focus on specific contexts—such as individual practice (McPherson and McCormick, 1999, 2000, 2006; Ritchie and Williamon, 2013; Araújo, 2016; Dos Santos Silva et al., 2024), practice and band/orchestra rehearsals (Austin and Berg, 2006; Miksza, 2012; Ersozlu and Miksza, 2015; Madeira et al., 2018), or practice and performance (Hatfield et al., 2017). They also adopt scales originally developed for general academic contexts, such as Pintrich and DeGroot’s (1990) *Motivated Strategies for Learning Questionnaire* (*MSLQ*) (McPherson and McCormick, 1999, 2000, 2006; Nielsen, 2004; Austin and Berg, 2006; Miksza, 2012) or generate items based on the theoretical framework of self-regulated learning (Miksza, 2012; Araújo, 2016; Hatfield et al., 2017). Because these top-down approaches may overlook behaviors unique to classical music performance, a new scale that addresses SRL in lessons, practice, and performance was developed using a bottom-up approach based on the above-mentioned case studies of elite classical musicians (Fujimoto and Uesaka, 2025).

Using these original scales, the study tested the hypothesis that the satisfaction of basic psychological needs in musical interpretation predicts a greater use of self-oriented interpretive approaches, which in turn predicts higher degrees of self-regulated learning behaviors, following the model of *Werktreue* internalization (Fujimoto and Uesaka, 2024).

## 2 Materials and methods

### 2.1 Materials

#### 2.1.1 Basic psychological need satisfaction in musical interpretation

To address basic psychological need satisfaction in musical interpretation, three items were developed, assessing the fulfillment of the needs for competence, autonomy, and relatedness, respectively (e.g., “I have the ability to understand the composer’s intentions”). All items were rated on a 5-point Likert scale, ranging from 1 (*strongly disagree*) to 5 (*strongly agree*).

#### 2.1.2 Self-oriented interpretive approaches

Six items were developed to address the six self-oriented interpretive approaches identified by Fujimoto and Uesaka (2024) (e.g., “I reflect my own emotions and individuality in my interpretation”). Respondents rated items on a scale ranging from 1 (*strongly disagree*) to 5 (*strongly agree*).

#### 2.1.3 Self-regulated learning in lessons, practice, and performance

A total of 31 items were generated to assess self-regulated learning behaviors in lessons, practice, and performance. Sixteen items were reverse-scored items, addressing other-regulated learning (ORL) behaviors (e.g., SRL in lessons (srl-l): “If I do not understand something during lessons, I ask questions”; ORL in lessons (orl-l): “I follow my teacher’s instructions even if I disagree with or do not understand them”; SRL in practice (srl-p): “I try out various musical expressions”; ORL in practice (orl-p): “I sometimes do not know what to practice; SRL in performance” (srl-pe): “I can be my true self during performance”; ORL in performance (orl-pe): “I rely on teachers’ or judges’ feedback to self-evaluate my performance”). Respondents rated items on a scale ranging from 1 (*strongly disagree*) to 5 (*strongly agree*).

Additional scales and open-ended questions were included in the survey, which will be reported in future studies.

### 2.2 Participants

Data were collected via Google Forms through snowball sampling, resulting in a total of 214 classical musicians (*M*_age_ = 23.48 years, *SD* = 6.25). The sample was predominantly female (79%), with four participants declining to report their gender. Participants represented a diverse range of specializations, including keyboard (37%), strings (35%), winds (19%), voice (7%), and percussion (1%). 23% were enrolled in music high schools, 35% were attending conservatories, and 42% were professional musicians, having graduated from conservatories. Two participants had not attended conservatories but had received private tuitions for an average of 15 years, participated in competitions, and performed public recitals regularly. While the majority of participants had received professional training in Japan, 6% were currently or had previously studied at music institutions abroad. Participants received an Amazon gift card of 500 Japanese yen upon completion of the survey. Ethical approval was obtained from the Research Ethics Committee at the University of Tokyo.

### 2.3 Analysis

Data analyses were conducted using R, version 2026.01.1 + 403. The significance level was set at *p* < 0.05 for all statistical tests.

After Harman’s single-factor test was performed using exploratory factor analysis with all items to assess the potential for common method bias, scales were validated through a series of Exploratory Factor Analysis (EFA) and Confirmatory Factor Analysis (CFA). The suitability of the data for factor analysis was assessed using the Kaiser-Meyer-Olkin (KMO) measure of sampling adequacy and Bartlett’s test of sphericity. An EFA was then conducted to evaluate the dimensionality of the scales. Robust maximum likelihood estimation was applied to account for any potential deviations from normality. Oblique rotation was used when multiple factors were extracted. Following recommendations by Stevens (2002), the cut-off point for factor loadings was set at 0.40. Additionally, items with cross-loadings exceeding 0.32 were considered for deletion (Costello and Osborne, 2005).

Subsequently, a CFA was performed with robust maximum likelihood estimation. Latent factors were permitted to correlate, while cross-loadings of items on unintended factors were constrained to zero. Model misspecification was identified by examining standardized residuals and modification indices. Goodness-of-fit was evaluated using the scaled chi-square (χ^2^), the robust Comparative Fit Index (CFI), the robust Tucker-Lewis Index (TLI), the robust Root Mean Square Error of Approximation (RMSEA), and the Standardized Root Mean Square Residual (SRMR). Since the chi-square (χ^2^) test is highly sensitive to sample size (Bentler and Bonett, 1980), values of CFI and TLI being close to 0.95 or greater, RMSEA being close to 0.06 or below, and SRMR being close to 0.08 or below were used as benchmarks for excellent model fit (Hu and Bentler, 1999; Brown, 2006). Once the model was finalized, internal consistency was evaluated based on the omega coefficient. The omega coefficient is preferred over Cronbach’s alpha when assumptions, such as tau-equivalence, normality, and uncorrelated error terms are violated (McNeish, 2018), as was the case in this study. Values between 0.65 and 0.80 are typically recommended in the exploratory stages of scale development (Kalkbrenner, 2023).

Following scale validation, a preliminary analysis was conducted to identify potential confounding variables: age, gender, and expertise level. Age was treated as a continuous variable, while gender and expertise level were dummy-coded. Given the non-normal distribution of BPNS-MI and SO-IA scores, Spearman’s rank correlation coefficient was used to assess their relationships with age, whereas Pearson’s product–moment correlation coefficient was applied to the normally distributed SRL-LPP scores. For gender comparisons, participants who declined to report their gender (*n* = 4) were excluded from the dataset, and differences between male and female groups were analyzed using the Mann–Whitney *U* test for BPNS-MI and SO-IA and a Welch independent samples *t*-test for SRL-LPP. Regarding expertise, participants were categorized into three levels: high school students, college students,^3^ and professionals. The Kruskal-Wallis test was used to examine differences in BPNS-MI and SO-IA scores across these levels, with Bonferroni-corrected Dunn’s post-hoc tests where significant effects were detected. For SRL-LPP scores, a one-way ANOVA was conducted, followed by Tukey’s Honestly Significant Difference (HSD) post-hoc tests where significant effects were found. Effect sizes were assessed using Cohen’s d for *t*-tests, *r* for Dunn’s tests, and $\eta^{2}\;$ for ANOVA and Kruskal-Wallis tests.

To test whether basic psychological need satisfaction in musical interpretation (BPNS-MI) predicts self-regulated learning behaviors (SRL-LPP) through the use of self-oriented interpretive approaches (SO-IA), mediation analysis was conducted with summed scores using the product of coefficients approach (MacKinnon, 2008). To address potential confounding variables (MacKinnon, 2008), age, gender, and expertise level were included as control variables, and robust maximum likelihood estimation was used to account for the non-normality of BPNS-MI and SO-IA scores. Participants who did not disclose their gender (*n* = 4) were excluded from the analysis, resulting in a final sample of *N* = 210.

## 3 Results

There was no missing data in the dataset. Although skewness fell within the absolute threshold of 2.0, indicating an acceptable level of normality (Curran et al., 1996), a robust maximum likelihood estimator was used to address potential non-normality. Descriptive statistics and factor loadings are displayed in Tables 1–3.

**Table 1:** Means, standard deviations, skewness, kurtosis, and factor loadings of the BPNS-MI items.

Item ID	BPNS-MI item	*M*	*SD*	Skewness	Kurtosis	Factor loading
bpns-mi-1	I have the ability to interpret the composer’s intentions.	2.7	1.09	0.14	−0.74	0.70
bpns-mi-2	I can connect with the audience through my interpretation.	3.57	1.22	−0.58	−0.64	0.64
bpns-mi-3	I choose my musical interpretation based on my own will.	4.02	1.04	−1.06	0.64	0.46

**Table 2:** Means, standard deviations, skewness, kurtosis, and factor loadings of the SO-IA items.

Item ID	SO-IA item	*M*	*SD*	Skewness	Kurtosis	Factor loading (EFA)	Factor loading (CFA)
so-ia-1	I reflect my own emotions and individuality in my interpretation.	3.88	1.07	−0.91	0.14	0.74	0.77
so-ia-2	I selectively adopt my teacher’s musical ideas to develop my own interpretation.	4.27	0.89	−1.06	0.23	0.56	0.55
so-ia-3	I value the composer’s intentions and emotions beyond the literal notation.	4.01	1.05	−1.12	0.68	0.49	0.49
so-ia-4	I do not think about interpretation much. (reverse-scored)	4.2	1.07	−1.31	0.88	0.48	0.46
so-ia-5	I change my interpretation spontaneously during performance.	2.63	1.39	0.36	−1.18	0.22	
so-ia-6	I always think about interpretation even when I am sight-reading or practicing technique.	3.03	1.42	0	−1.39	0.24	

**Table 3:** Means, standard deviations, skewness, kurtosis, and factor loadings of the SRL-LPP items.

Item ID	SRL-LPP item	*M*	*SD*	Skewness	Kurtosis	Factor loading				
						1	2	3	4	5
Factor 1: musical goal setting										
srl-pe-12	I aim to communicate my expression in performance.	4.43	0.85	−1.92	4.23	**0.61**	0.17	0.01	0.04	0.14
srl-p-1	When I start learning a piece, I think about what kind of performance I want to give.	3.74	1.15	−0.77	−0.27	**0.50**	0.03	0.04	0.05	0.35
srl-p-2	I analyze the score.	3.73	1.17	−0.73	−0.33	**0.46**	0.05	0.04	0.10	0.19
srl-pe-11	I have a clear mental image of my playing before a performance.	4.03	1.1	−1.13	0.50	**0.46**	0.09	0.07	0.04	0.01
srl-p-4	I listen to different recordings to find my own way of expressing the music.	3.88	1.25	−0.98	−0.08	**0.44**	0.04	0.05	0.10	0.16
srl-p-3	I try out various musical expressions.	3.85	1.14	−0.8	−0.36	**0.42**	0.04	0.01	0.01	0.28
Factor 2: practice engagement										
orl-p-2	I do not feel motivated to practice.	2.85	1.27	0.13	−1.02	0.04	**1.01**	0.05	0.10	0.08
orl-p-3	I cannot concentrate on practice.	2.94	1.25	0.01	−1.08	0.09	**0.61**	0.02	0.02	0.17
srl-p-6	I enjoy practicing.	3.17	1.23	−0.18	−0.95	0.19	**0.50**	0.14	0.09	0
Factor 3: active lesson participation										
srl-l-7	If I do not understand something during lessons, I ask questions.	4.35	0.92	−1.46	1.61	0.03	0.08	**0.89**	0.24	0.04
orl-l-8	I cannot express my opinions to my teacher.	3.87	1.13	−0.83	−0.27	0.02	0.02	**0.78**	0.03	0.10
srl-l-8	I can perform the way I want during lessons.	3.21	1.2	−0.13	−0.99	0.19	0.06	**0.32**	0.28	0.18
Factor 4: psychological performance optimization										
orl-pe-14	Because I get nervous, I cannot perform well.	2.5	1.26	0.42	−0.95	0.03	0.12	0.15	**0.81**	0.04
orl-pe-12	I feel anxious about my playing before performance.	2.07	1.25	0.98	−0.18	0.29	0.07	0.08	**0.70**	0.05
orl-pe-16	I experience negative emotions (e.g., regret, shame, or guilt) after performance.	2.98	1.4	−0.02	−1.31	0.03	0.02	0	**0.69**	0.02
srl-pe-13	I can be my true self during performance.	3.24	1.26	−0.2	−1.13	0.07	0.04	0.06	**0.65**	0.06
orl-l-7	I get nervous and cannot perform as intended during lessons.	2.71	1.27	0.25	−1.16	0.14	0.02	0.25	**0.52**	0.13
srl-pe-15	I feel satisfied with my playing after performance.	2.86	1.14	0.04	−0.87	0.20	0.01	0.02	**0.47**	0.11
orl-pe-13	I struggle to immerse myself in the music during performance.	3.64	1.15	−0.64	−0.49	0.08	0.06	0.03	**0.43**	0.07
srl-pe-14	I adapt flexibly to the changing moments and situations during performance.	3.64	1.21	−0.64	−0.54	0.31	0.12	0.12	**0.37**	0.21
Factor 5: independence from teachers										
orl-l-9	I rely on my teacher for expressive choices.	3.19	1.28	0	−1.19	0.06	0.14	0.04	0.02	**0.66**
orl-p-4	I only practice what my teacher told me.	4	1.05	−0.92	0.1	0.23	0.01	0.07	0.11	**0.65**
orl-p-5	I work on expression only after I can play the piece technically.	2.83	1.44	0.11	−1.39	0.11	0.12	0.05	0.09	**0.44**
orl-p-1	I sometimes do not know what to practice.	3.38	1.29	−0.36	−1.02	0.06	0.19	0.05	0.10	**0.42**
orl-pe-15	I rely on teachers’ or judges’ feedback to self-evaluate my performance.	3	1.18	0.11	−0.98	0.05	0.04	0.08	0.13	**0.39**
Unused items										
srl-p-5	I feel satisfied with my practice sessions.	2.82	1.19	0.07	−1.01	0.14	0.04	0.04	0.36	0.17
srl-l-9	I selectively incorporate my teacher’s instructions to my own practice.	3.99	1.19	−1.11	0.18	0.25	0.09	0.12	0.08	0.13
srl-l-10	I enjoy taking lessons.	4.32	0.94	−1.48	1.86	0.28	0.16	0.20	0.08	0.09
orl-p-6	I practice for long hours due to anxiety.	2.9	1.44	0.09	−1.38	0.27	0.21	0.06	0.33	0.19
orl-l-10	I follow my teacher’s instructions even if I disagree with or do not understand them.	3	1.18	−0.04	−0.92	0.11	0.09	0.25	0.09	0.17
orl-pe-11	I fixate on a single way of performing before a performance.	2.94	1.35	0.08	−1.23	0.37	0.02	0.01	0.07	0.19

Srl-l, SRL in lessons; orl-l, ORL in lessons; srl-p, SRL in practice; orl-p, ORL in practice; srl-pe, SRL in performance; orl-pe, ORL in performance. Items for ORL are reverse-scored. Items that were later incorporated into the CFA model are also listed under each factor.Bold values indicate primary factor loadings for the measurement model.

Harman’s single-factor test indicated that a single factor accounted for only 14.58% of the total variance, which is well below the 50% threshold. This suggests that common method bias is unlikely to be a significant concern in the current study.

### 3.1 Scale validation

#### 3.1.1 Basic psychological need satisfaction in musical interpretation

The Kaiser-Meyer-Olkin (KMO) measure of sampling adequacy was sufficient (*KMO* = 0.625), and Bartlett’s test of sphericity confirmed that the data were suitable for factor analysis ( $\chi^{2}$ (3) = 76.85, *p* < 0.001). As the three items were theoretically assumed to represent basic psychological need satisfaction in music interpretation, a CFA was conducted using a one-factor model. Because the scale consisted of only three items, the model was just identified. The average variance extracted (AVE) was 0.38, and the omega coefficient was 0.64.

#### 3.1.2 Self-oriented interpretive approaches

The Kaiser-Meyer-Olkin (KMO) measure indicated acceptable sampling adequacy (*KMO* = 0.685), and Bartlett’s test of sphericity confirmed the suitability for factor analysis ( $\chi^{2}$ (15) = 144.33, *p* < 0.001). Based on results of a parallel analysis, an EFA was conducted using a one-factor model. The analysis indicated that the factor explained 24% of the variance in the data, and some of the model fit indices remained marginal ( $\chi^{2}$ (9) = 19.452, *p* = 0.02, robust CFI = 0.92, robust TLI = 0.86, robust RMSEA = 0.074, 90% CI [0.027, 0.120], SRMR = 0.05).

Two items addressing the improvisatory and integrated approaches, so-ia-5 (“I change my interpretation spontaneously during performance”) and so-ia-6 (“I always think about interpretation, even when sight-reading or practicing technique”), had low factor loadings of 0.22 and 0.24. Post-hoc pairwise Wilcoxon signed-rank tests revealed that scores for these two items were significantly lower than all other interpretive behaviors (*p* < 0.001), with the improvisatory approach (so-ia-5) yielding the lowest mean (*M* = 2.63). These results suggest that these items represent highly specialized or less conscious behaviors that diverge from other interpretive approaches. Thus, a CFA was conducted excluding the two items; the resulting fit indices indicated excellent fit ( $\chi^{2}$ (6) = 4.262, *p* = 0.12, robust CFI = 0.97, robust TLI = 0.93, robust RMSEA = 0.078, 90% CI [0.000, 0.183], SRMR = 0.035). The average variance extracted (AVE) was 0.34, and the omega coefficient was 0.66.

While the internal consistency for BPNS-MI and SO-IA is marginal, as this study represents a first attempt to measure interpretive autonomy from both a psychological and behavioral perspective with a small set of items, the reliability of these two scales was considered permissible for subsequent analyses.

#### 3.1.3 Self-regulated learning in lessons, practice, and performance

The Kaiser-Meyer-Olkin (KMO) measure of sampling adequacy was acceptable (*KMO* = 0.752), and Bartlett’s test of sphericity also supported that the data were suitable for factor analysis ( $\chi^{2}$ (465) = 1789.54, *p* < 0.001). Following parallel analysis, an EFA was conducted for a five-factor model. The factors accounted for 35% of the total variance explained. Although item srl-p-1 (“When I start learning a piece, I think about what kind of performance I want to give”) exhibited a secondary loading of 0.35 on Factor 5, it was retained because the ratio of the squared primary loading to the squared secondary loading was 2.04. According to the guideline by Hair et al. (2018), this cross-loading can be ignored for interpretation purposes, as the ratio exceeds the threshold of 2.0.

Initial CFA results revealed a Heywood case, primarily due to Factor 3 being under-identified with only two indicators. To resolve this, item srl-l-8, which exhibited the factor loading of 0.32 in EFA, was incorporated into the factor (*λ* = 0.38). The model, however, still demonstrated suboptimal fit ( $\chi^{2}$ (225) = 370.536, *p* < 0.001, robust CFI = 0.85, robust TLI = 0.83, robust RMSEA = 0.06, 90% CI [0.05, 0.07], SRMR = 0.077). Examination of Bentler’s standardized residual covariances revealed a high value (0.45) between the residuals of srl-l-8 (“I can perform the way I want during lessons”) and orl-l-7 (“I get nervous and cannot perform as intended during lessons”), indicating significant local misfit. Deleting item orl-l-7 improved the model fit ( $\chi^{2}$ (204) = 292.853, *p* < 0.001, robust CFI = 0.90, robust TLI = 0.89, robust RMSEA = 0.05, 90% CI [0.04, 0.06], SRMR = 0.071). Additionally, item srl-p-4 was removed from Factor 1 due to a decreased loading of 0.22. Items with factor loadings above 0.40 in the CFA model were then added: srl-pe-14 was added to Factor 4 and item orl-pe-15 was added to Factor 5. Finally, a correlation between the residuals of items orl-pe-12 (“I feel anxious about my playing before performance”) and orl-pe-14 (“Because I get nervous, I cannot perform well”) was specified (*r* = 0.32, *p* = 0.001), as both items may be influenced by high levels of music performance anxiety. Following these modifications, the model showed acceptable fit ( $\chi^{2}$ (224) = 305.197, *p* < 0.001, robust CFI = 0.91, robust TLI = 0.90, robust RMSEA = 0.04, 90% CI [0.03, 0.06], SRMR = 0.07). Factor loadings for the final CFA model are presented in Table 4.

**Table 4:** Factor loadings from the final confirmatory factor analysis (CFA) for the SRL-LPP scale.

Item ID	SRL-LPP item	Factor loading				
		1	2	3	4	5
Factor 1: musical goal setting						
srl-p-1	When I start learning a piece, I think about what kind of performance I want to give.	0.62				
srl-p-2	I analyze the score.	0.50				
srl-p-3	I try out various musical expressions.	0.50				
srl-pe-11	I have a clear mental image of my playing before a performance.	0.47				
srl-pe-12	I aim to communicate my expression in performance.	0.47				
Factor 2: practice engagement						
orl-p-2	I do not feel motivated to practice.		0.92			
orl-p-3	I cannot concentrate on practice.		0.62			
srl-p-6	I enjoy practicing.		0.57			
Factor 3: active lesson participation						
orl-l-8	I cannot express my opinions to my teacher.			0.93		
srl-l-7	If I do not understand something during lessons, I ask questions.			0.68		
srl-l-8	I can perform the way I want during lessons.			0.38		
Factor 4: psychological performance optimization						
orl-pe-14	Because I get nervous, I cannot perform well.				0.68	
srl-pe-13	I can be my true self during performance.				0.67	
orl-pe-16	I experience negative emotions (e.g., regret, shame, or guilt) after performance.				0.64	
orl-pe-13	I struggle to immerse myself in the music during performance.				0.58	
orl-pe-12	I feel anxious about my playing before performance.				0.52	
srl-pe-14	I adapt flexibly to each moment and situation during performance.				0.48	
srl-pe-15	I feel satisfied with my playing after performance.				0.47	
Factor 5: independence from teachers						
orl-l-9	I rely on my teacher for expressive choices.					0.69
orl-p-4	I only practice what my teacher told me.					0.66
orl-p-1	I sometimes do not know what to practice.					0.52
orl-p-5	I work on expression only after I can play the piece technically.					0.43
orl-pe-15	I rely on teachers’ or judges’ feedback to self-evaluate my performance.					0.42

Srl-l, SRL in lessons; orl-l, ORL in lessons; srl-p, SRL in practice; orl-p, ORL in practice; srl-pe, SRL in performance; orl-pe, ORL in performance. Items for ORL are reverse-scored.

The five subscales were labeled based on items as following: Musical Goal Setting, Practice Engagement, Active Lesson Participation, Psychological Performance Optimization, and Independence from Teachers. The hierarchical omega total ( $\omega_{h}$ = 0.74) indicated an acceptable level of reliability for the overarching construct. For the subscales, the reliability ranged from 0.64 to 0.77, and the average variance extracted (AVE) ranged from 0.27 to 0.52; they were considered satisfactory, given that this study represents an initial attempt to measure self-regulated learning behaviors across lessons, practice, and performance. All the factors were positively inter-correlated (*r* = 0.19 to 0.55), and each factor contributed significantly to the higher-order SRL-LPP construct with factor loadings ranging from 0.41 to 0.75 (Table 5). These results support the existence of an overarching psychological construct of self-regulation behind learning behaviors across lesson, practice, and performance contexts.

**Table 5:** Factor correlations and internal consistency for the SRL-LPP scale.

Scales	1	2	3	4	5	Omega coefficient
1. Musical goal setting	ー					0.64
2. Practice engagement	0.35	ー				0.76
3. Active lesson participation	0.31	0.19	ー			0.71
4. Psychological performance optimization	0.39	0.24	0.21	ー		0.77
5. Independence from teachers	0.55	0.34	0.30	0.38	ー	0.67
6. SRL-LPP	0.75	0.46	0.41	0.52	0.73	0.74

SRL-LPP, Self-regulated learning in lessons, practice, and performance.

### 3.2 Preliminary analysis

Preliminary analyses were conducted to identify potential confounding variables by examining whether scores on BPNS-MI, IA, and SRL-LPP differed by age, gender, or expertise level.

#### 3.2.1 Basic psychological need satisfaction in musical interpretation

The BPNS-MI score ranged from 3 to 15 with a mean of 10.29 (*SD* = 2.53), exhibiting a slight negative skew (−0.47) and a kurtosis of −0.16. A Shapiro–Wilk test confirmed that the distribution significantly deviated from normality, *W* = 0.97, *p* < 0.001. Consequently, non-parametric tests were employed for subsequent analyses.

No significant differences were found across age (*r* = 0.13, *p* = 0.057), gender (*W* = 3066.5, *p* = 0.19), or expertise level ( $\chi^{2}$ (2) = 4.40, *p* = 0.11). This suggests that basic psychological need satisfaction in musical interpretation is consistent across these variables within this sample.

#### 3.2.2 Self-oriented interpretive approaches

The SO-IA score ranged from 6 to 20 with a mean of 16.36 (*SD* = 2.85). The distribution exhibited a negative skew (−0.96) and a kurtosis of 0.85. A Shapiro–Wilk test confirmed that the distribution significantly deviated from normality, *W* = 0.92, *p* < 0.001. Consequently, non-parametric tests were employed for subsequent analyses.

There were small but significant effects for age (*r* = 0.15, *p* = 0.02) and expertise level ( $\chi^{2}$ (2) = 7.79, *p* = 0.02, $\eta^{2}\;$ = 0.027), while no gender differences were observed (*W* = 2,954, *p* = 0.10). High school students (*M* = 15.40, *SD* = 3.04) reported less use of self-oriented interpretive approaches than both college students (*M* = 16.84, *SD* = 2.18, *p* = 0.036, *r* = 0.17) and professionals (*M* = 16.49, *SD* = 3.12, *p* = 0.036, *r* = 0.17). No significant difference was found between college students and professionals (*p* = 1.00).

#### 3.2.3 Self-regulated learning in lessons, practice, and performance

Self-Regulated Learning in Lessons, Practice, and Performance (SRL-LPP) scores ranged from 37 to 111 with a mean of 77.5 (*SD* = 12.2). The scores had an approximately normal distribution (skewness = −0.17, kurtosis = −0.24), and a Shapiro–Wilk test confirmed that the data did not significantly deviate from normality, *W* = 0.99, *p* = 0.27.

For the overall SRL-LPP scale, no significant differences were found across gender (*t*(68.4) = −1.49, *p* = 0.14) or expertise level (*F*(2, 211) = 2.10, *p* = 0.13). However, significant differences were found for the Independence from Teachers subscale; male musicians (*M* = 18.24, *SD* = 3.80) reported higher levels of independence than female musicians (*M* = 16.04, *SD* = 4.02), *t*(65.9) = 3.31, *p* = 0.002, d = 0.56. College students (*M* = 16.86, *SD* = 3.70) and professionals (*M* = 17.04, *SD* = 4.24) reported greater independence than high school students (*M* = 14.52, *SD* = 3.94), *F*(2, 211) = 7.22, *p* < 0.001, $\;\eta^{2}$ = 0.064.

There was a weak positive relationship between overall SRL-LPP and age, *r* = 0.14, *p* = 0.047. For the subscales, age was negatively associated with Active Lesson Participation (*r* = −0.15, *p* = 0.02) but positively associated with Independence from Teachers (*r* = 0.25, *p* < 0.001). This implies that while older musicians may engage less actively during lessons, they have higher levels of independence from their teachers outside the lessons.

### 3.3 Mediation analysis

Summed scores of the three scales were moderately correlated with each other, as shown in Table 6, which presents the Spearman’s rank correlation coefficients among the BPNS-MI, SO-IA, and SRL-LPP scales. Mediation analysis was conducted to test the hypothesized model, where basic psychological need satisfaction in musical interpretation (BPNS-MI) predicts the use of self-oriented interpretive approaches (SO-IA), which then predicts self-regulated learning behaviors (SRL-LPP). Following the results from the preliminary analysis, age and expertise level were included as predictors of SO-IA, and age, gender, and expertise level were included as predictors of SRL-LPP.

**Table 6:** Correlations between BPNS-MI, SO-IA, and SRL-LPP.

Variables	1	2	3	4	5	6	7
1. BPNS-MI	ー						
2. SO-IA	0.38***	ー					
3. SRL-LPP	0.48***	0.38***					
4. Musical goal setting	0.42***	0.39***	0.62***				
5. Practice engagement	0.13*	0.18**	0.54***	0.27***			
6. Active lesson participation	0.23***	0.21**	0.52***	0.23***	0.24***		
7. Psychological performance optimization	0.31***	0.18**	0.73***	0.25***	0.21**	0.28***	
8. Independence from teachers	0.39***	0.32***	0.66***	0.31***	0.25***	0.16*	0.32***

*p <.05, **p <.01, ***p <.001; BPNS-MI, Basic psychological need satisfaction in musical interpretation; SO-IA, Self-oriented interpretive approaches; SRL-LPP, Self-regulated learning in lessons, practice, and performance.

**Figure 1:**
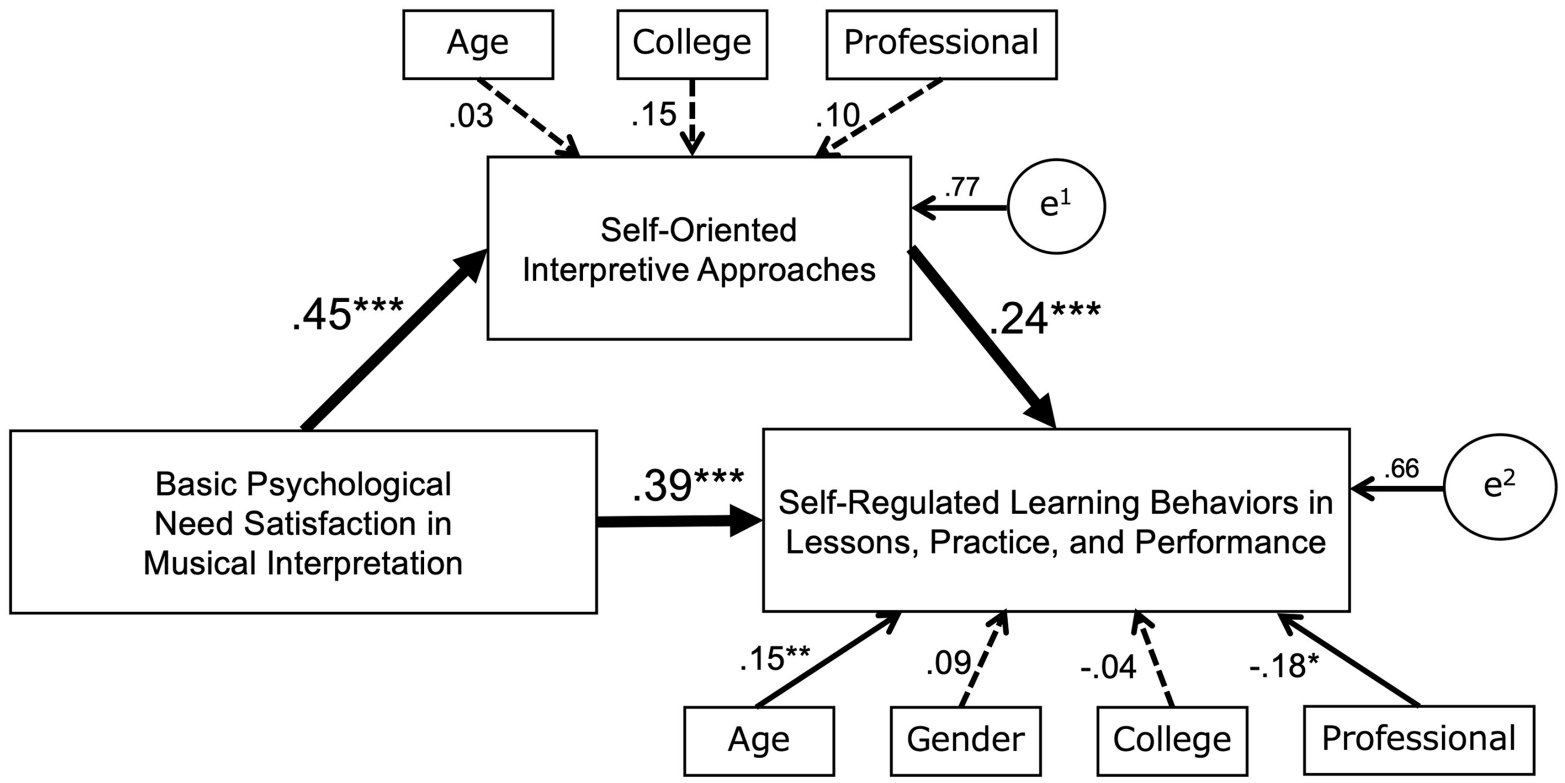
Mediation model of BPNS-MI predicting SRL-LPP, partially mediated by SO-IA. *p < 0.05, **p < 0.01, ***p < 0.001. Significant paths are represented by solid lines, and non-significant paths are represented by dashed lines.

The analysis supported the hypothesis; BPNS-MI positively predicted SO-IA ( β = 0.45, *p* < 0.001), which in turn predicted higher levels of SRL-LPP ( β = 0.24, *p* = 0.001). The indirect effect through SO-IA was statistically significant ( β = 0.11, *p* = 0.004), accounting for 21.7% of the total effect. The direct effect of BPNS-MI on SRL-LPP was also significant ( β = 0.39, *p* < 0.001), and the total effect of BPNS-MI on SRL-LPP was large ( β = 0.49, *p* < 0.001). Among the control variables, significant effects were observed only for age ( β = 0.15, *p* = 0.004) and expertise level; notably, professional status was negatively associated with SRL-LPP ( β = − 0.18, *p* = 0.04), indicating slightly lower reported self-regulation among professionals compared to students. Overall, the model demonstrated excellent fit ( $\chi^{2}$ (1) = 1.024, *p* = 0.31, robust CFI = 1.00, robust TLI = 0.998, robust RMSEA = 0.01, 90% CI [0.000, 0.165], SRMR = 0.011) and accounted for 34% of the variance in self-regulated learning behaviors (Figure 1).

To confirm the several assumptions for mediation models (MacKinnon, 2008), an interaction term between BPNS-MI and SO-IA was introduced into the model to test for potential moderation. The interaction effect was non-significant ( β = 0.13, *p* = 0.77) and its inclusion substantially degraded model fit (e.g., CFI = 0.238), indicating that the effect of BPNS-MI on SRL-LPP was not moderated by SO-IA. In addition, a series of linear mixed-effects models confirmed that the mediated effects were highly consistent across age, gender, and expertise level, as the standardized coefficients remained within a narrow range (BPNS-MI on SO-IA: 0.43 to 0.46; SO-IA on SRL-LPP: 0.23 to 0.25; BPNS-MI on SRL-LPP: 0.38 to 0.39). These findings suggest that the mediation process is additive and applies to classical musicians regardless of age, gender, and expertise level.

## 4 Discussion

This is the first study that quantitatively examined how interpretive autonomy predicts classical musicians’ self-regulated learning behaviors across lesson, practice, and performance contexts. The study found that the satisfaction of basic psychological needs in musical interpretation predicted self-regulated learning behaviors, both directly and indirectly through the use of self-oriented interpretive approaches. Importantly, the mechanism remained robust regardless of age, gender, and expertise level. The findings support the model of *Werktreue* internalization (Fujimoto and Uesaka, 2024), which proposed the definition, causes, and effects of interpretive autonomy by applying self-determination theory (Deci and Ryan, 2000). They are also in line with qualitative studies which show that music students with higher interpretive autonomy demonstrated higher levels of self-regulation in lessons, practice, and performance (e.g., Reid, 2001; McPherson et al., 2019; Fujimoto and Uesaka, 2025).

In addition, this study represents the first attempt to measure interpretive autonomy—which has long been discussed conceptually by musicologists and musicians (e.g., Taruskin, 1995; Doğantan-Dack, 2017; Leech-Wilkinson, 2020)—by addressing both its psychological and behavioral aspects. While the scales would benefit from further refinement, they can significantly advance quantitative studies on interpretive autonomy.

Two removed items that addressed the improvisatory and integrated approaches in the SO-IA scale had lower means than other items, suggesting that these approaches are employed less frequently or are less consciously. The improvisatory approach, where musicians spontaneously generate interpretive ideas during performance, is less common in current classical performance practice (Hill, 2017; Dolan et al., 2018). Similarly, the integrated approach, in which a musician considers interpretive ideas while sight-reading or working on technical aspects, may be employed unconsciously by advanced musicians (Hallam, 1995; Chaffin and Imreh, 2001; Wise et al., 2017) and thus may not be easily captured by self-report. Nevertheless, because these approaches are characteristic of highly autonomous musicians (Holmes, 2005; Silverman, 2008; Hill, 2017), further research is needed to refine the scale to understand how these approaches contribute to the latent construct.

The Self-Regulated Learning in Lesson, Practice, and Performance (SRL-LPP) scale distinguishes itself from existing SRL measures by comprehensively addressing learning behaviors across lesson, practice, and performance contexts. The second-order model, comprising five factors, supports the existence of a generalized self-regulation skill that governs learning behaviors across different learning environments. If students show maladaptive behaviors in one setting, such as passivity during lessons or emotional dysregulation during performance, they are likely to demonstrate a lack of self-regulation during solitary practice.

Furthermore, the study revealed a wide range of levels of interpretive autonomy and self-regulation among both student and professional musicians. This supports the controversial argument that some classical musicians lack autonomy when developing original interpretations (Taruskin, 1995; Leech-Wilkinson, 2020) and fail to initiate self-regulation (Dos Santos Silva and Marinho, 2025). It also implies that the “cultivation of a personal artistic voice could be given lower priority” in professional music education (Doğantan-Dack, 2017, p. 133), highlighting the need to investigate how professional education nurtures artists within a complex cultural context.

Lastly, several limitations need to be noted. First, as this study represents a preliminary attempt to measure basic psychological need satisfaction in musical interpretation (BPNS-MI), self-oriented interpretive approaches (SO-IA), and self-regulated learning across three learning contexts (SRL-LPP), further research is required to cross-validate and refine these scales using independent samples. In addition to the limited number of items per factor, the bandwidth-fidelity dilemma may have impacted the internal consistency (Cronbach and Gleser, 1957), given that the items addressed broad psychological states, cognitive strategies, and learning behaviors. In addition, while the present study addressed several assumptions underlying mediation models, causal inferences cannot be drawn from this cross-sectional study, and the direction of paths is assumed *a* *priori* by the researcher following the theoretical proposition. Moreover, the analysis was based on summed scores rather than latent variables, and future studies may account for measurement error. Finally, the findings may be subject to cultural influences specific to the Japanese context, as well as the inherent biases of self-reported data.

## 5 Conclusion

This study demonstrated that interpretive autonomy significantly predicts self-regulated learning behaviors across lessons, practice, and performance. Specifically, the findings revealed that musicians with higher basic psychological need satisfaction in musical interpretation were more likely to employ self-oriented interpretive approaches, which in turn predicted greater self-regulated learning behaviors. Furthermore, the study provided preliminary evidence that interpretive autonomy can be quantified from both psychological and behavioral aspects using psychometric scales. This significantly advances the discourse on interpretive autonomy that has long been explored primarily through musicological and qualitative studies. The findings also revealed varied levels of interpretive autonomy and self-regulation among both students and professionals. This raises a critical question regarding how to foster interpretive autonomy to fulfill the complex goal of professional music education, which entails nurturing musicians who can articulate a creative, artistic voice while critically engaging with, negotiating, and potentially transforming established norms. Future research should investigate the learning environments and interpersonal dynamics that either facilitate or impede the development of interpretive autonomy within professional training.

## Data Availability

The raw data supporting the conclusions of this article will be made available by the authors, without undue reservation.

## Footnotes

1 Because autonomous or controlled *Werktreue* internalization is conceptualized solely based on basic psychological need satisfaction in musical interpretation, it does not explain what view a musician holds regarding the *Werktreue* ideology, such as their degree of deviation from the score or performance practices. It also does not explain how critically a musician reflects on or challenges the *Werktreue* ideology.

2 While self-oriented interpretive approaches may seem to reflect a subjective view (Silverman, 2007), the case studies by Fujimoto and Uesaka (2025) confirmed that self-oriented interpretive approaches are employed regardless of musicians’ views regarding the *Werktreue* ideology, when basic psychological needs are satisfied in musical interpretation.

3 The participants’ academic levels ranged from Bachelor of Music to Doctor of Musical Arts; however, those currently enrolled in a music college or conservatory were categorized as “college students” regardless of their degree levels.
